# Connecting to the long axis

**DOI:** 10.7554/eLife.83718

**Published:** 2022-11-08

**Authors:** Bryan A Strange

**Affiliations:** 1 Laboratory for Clinical Neuroscience, Center for Biomedical Technology, University Politécnica de Madrid Madrid Spain; 2 Reina Sofia Centre for Alzheimer's Research Madrid Spain

**Keywords:** hippocampus, structural connectivity, diffusion-weighted imaging, track-weighted imaging, hippocampal subfields, Human

## Abstract

New study reveals how various regions of the human cortex connect to the hippocampus along its longer anterior-posterior axis, shedding light on the way this structure is functionally organized.

**Related research article** Dalton MA, D’Souza A, Lv J, Calamante F. 2022. New insights into anatomical connectivityalong the anterior–posterior axis ofthe human hippocampus using in vivoquantitative fibre tracking. *eLife* **11**:e76143. doi: 10.7554/eLife.76143.

Few questions in neuroscience generate as much debate and interest as: “what does the hippocampus do?”. This small, cylinder-like structure is embedded in each brain hemisphere, where it participates in spatial navigation, episodic memory, emotional responses and other cognitive functions. Much of what is known about the hippocampus has come from studying the organization of a complex neural circuit along its short, transverse axis (Marr, 1971). However, a promising avenue to understand how the hippocampus can accommodate its multiple functions is to study its long axis, which extends from its head (anterior) to its tail (posterior).

Reports showing the rodent equivalent of the hippocampal head and tail carrying out distinct roles can be traced back over 50 years (Nadel, 1968). Furthermore, differences in neural activity have been observed across the anterior-posterior axis of the human brain at rest and during task-based activities (Vogel et al., 2020; Strange et al., 1999). Recent studies have shown that distinct regions along the long axis of the hippocampus may control specific cognitive behaviours (Strange et al., 2014). Yet, little is known about how this functional organisation is tied to anatomical connections in the human brain – that is, how areas along the anterior-posterior axis connect to the distant cortical regions associated with the relevant cognitive process. Now, in eLife, Marshall Dalton and colleagues from the University of Sydney report new findings that help to answer this question (Dalton et al., 2022).

The team used a quantitative analysis protocol to analyse images of the human brain captured using diffusion-weighted magnetic resonance imaging (Smith et al., 2015). This non-invasive technique provided a biologically meaningful estimate of the number of nerve fibres, or connectivity, between the hippocampus and various areas in the cortex. Dalton et al. also refined the approach to see where fibres from these distant cortical regions preferentially projected to within the hippocampus.

This revealed that the posterior hippocampus has multiple connections with the primary and early visual cortex and the medial parietal cortex, and this connectivity gradually decreases towards the head of the hippocampus. By contrast, the anterior hippocampus was more strongly linked to the temporal pole and lateral temporal cortex, with only a small number of nerve fibres from these cortical areas projecting to the hippocampal tail. Some regions in the hippocampus received inputs from multiple cortical areas. This is in keeping with other models which also show discrete areas of connectivity between the hippocampus and specific brain areas, and other cortical connections which gradually change in density further along the anterior-posterior axis (Strange et al., 2014; Figure 1).

**Figure 1.**
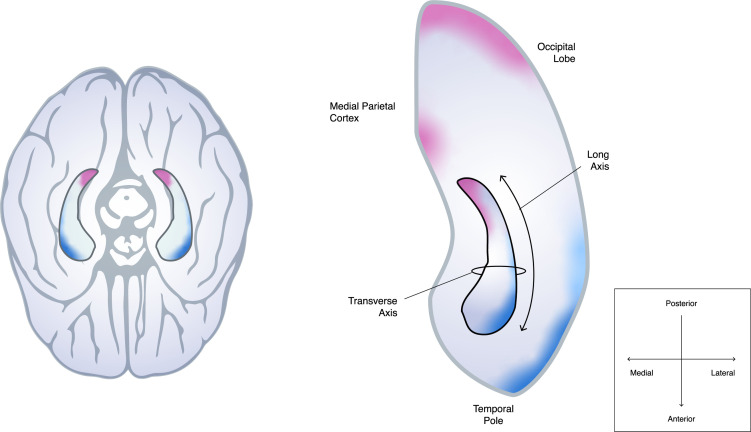
Schematic of how the hippocampus connects to different parts of the cortex along its anterior-posterior axis. Dalton et al. studied how the hippocampus, a cylinder-like structure in both hemispheres of the brain (left, viewed from below), connects to other parts of the cortex: the left hippocampus (outlined in black) and the brain regions surrounding it are shown on the right-hand side. It is well established that parts of the hippocampal transverse axis – which extends from the middle (medial) to the edge (lateral) of the brain – are connected to nearby parts of the cortex involved in cognition. However, Dalton et al. found that more distant cortical regions also anatomically connect to distinct regions along the long axis of the hippocampus, which spans from the anterior to posterior of the brain: nerve fibres in the occipital and medial parietal cortex (regions highlighted in pink) primarily project to the medial side of the posterior hippocampus, whereas the temporal pole and lateral temporal cortex (highlighted in blue) are more strongly linked to the lateral side of the anterior hippocampus. The density of these connections then gradually changes from the head to the tail of the hippocampus. There are also discrete areas of high connectivity which are not shown in the figure.

Image credit: Ramón Ruiz-Alba, Genco Pura Studio (CC BY 4.0)

Dalton et al. found that the connection between the hippocampus and the visual system is much stronger in humans than in rodents (the species from which most insights about the anterior-posterior axis have derived). Emerging evidence suggests that, in primates, the homologues of neuronal activity related to spatial self-location in rodents (e.g. place or grid cell activity) may in fact be view or grid-like responses to visual space (Nau et al., 2018; Rolls and Wirth, 2018). Thus, the finding of Dalton et al. that the human visual cortex and hippocampus are more strongly connected than has been appreciated in rodents is an important discovery. Furthermore, connections with the early visual cortex were higher in the posterior hippocampus, a section which, in primates, is thought to have a more prominent role in spatial navigation than the anterior portion. As certain regions in the early visual cortex receive inputs from particular parts of the retina, the findings from Dalton et al. raise the intriguing question of whether this ‘retinotopic’ information is also relayed to the posterior hippocampus.

As with all studies, there are some limitations. For instance, it is possible that the connections between the hippocampus and the visual areas which Dalton et al. report are, at least in part, a fusion of fibres within a well-known tract that links the visual cortex to a structure very close to the body and tail of the hippocampus. Furthermore, some of the results of Dalton et al. differ from non-human primate studies (Insausti and Muñoz, 2001), which could be due to limitations of non-invasive neuronal tracing methods in humans, rather than legitimate differences between species.

Dalton et al. also found that the density of neuron ‘ends’ varied across the transverse axis of the hippocampus. For instance, nerve fibres projecting from the visual and parietal cortex (which process visual and sensory information, respectively) seemed to mostly terminate on the medial region of the posterior hippocampus; whereas, neurons in the temporal lobe (which processes semantic information) tended to connect to the lateral side of the anterior hippocampus. However, it is unclear why this might be and how it impacts cognitive functions – although some clues may emerge from our understanding of the hippocampal transverse axis. It will be important to determine how the hippocampus integrates information from these various cortical areas during cognitive processes, like recalling a memory, given that they project to different places.

Another surprising finding of this study is the relatively low connectivity between the anterior portion of the hippocampus and the medial prefrontal cortex, as this connection is important for a number of brain functions (Barnett et al., 2021), such as forming integrated memories from overlapping experiences (Zeithamova et al., 2012). However, Dalton et al. mention that there is evidence to suggest that the number of nerve fibres directly projecting between the prefrontal cortex and hippocampus is quite sparse in the human brain. Nevertheless, this raises a more fundamental question regarding the extent that anatomically observed connections reflect functional relationships between regions, and whether even a small number of neurons can strongly couple parts of the brain together. Future work using the imaging method adopted by Dalton et al. will be important for answering this question.
